# Forest Restoration in a Fog Oasis: Evidence Indicates Need for Cultural Awareness in Constructing the Reference

**DOI:** 10.1371/journal.pone.0023004

**Published:** 2011-08-02

**Authors:** Luís Balaguer, Rosa Arroyo-García, Percy Jiménez, María Dolores Jiménez, Luís Villegas, Irene Cordero, Rafael Rubio de Casas, Raúl Fernández-Delgado, María Eugenia Ron, Esteban Manrique, Pablo Vargas, Emilio Cano, José J. Pueyo, James Aronson

**Affiliations:** 1 Departamento de Biología Vegetal I, Universidad Complutense de Madrid, Madrid, Spain; 2 Departamento de Biotecnología, Instituto Nacional de Investigación y Tecnología Agraria y Alimentaria, INIA, Madrid, Spain; 3 IRECA, Universidad Nacional de San Agustín, Arequipa, Peru; 4 Departamento Interuniversitario de Ecología, Universidad Complutense de Madrid, Madrid, Spain; 5 Instituto de Ciencias Agrarias, Consejo Superior de Investigaciones Científicas, Madrid, Spain; 6 National Evolutionary Synthesis Center (NESCent), Durham, North Carolina, United States of America; 7 Real Jardín Botánico de Madrid, Consejo Superior de Investigaciones Científicas, Madrid, Spain; 8 Centre d'Ecologie Fonctionelle et Evolutive, (C.N.R.S.-U.M.R. 5175), Montpellier, France; 9 Missouri Botanical Garden, St. Louis, Missouri, United States of America; Purdue University, United States of America

## Abstract

**Background:**

In the Peruvian Coastal Desert, an archipelago of fog oases, locally called lomas, are centers of biodiversity and of past human activity. Fog interception by a tree canopy, dominated by the legume tree tara (*Caesalpinia spinosa*), enables the occurrence in the Atiquipa lomas (southern Peru) of an environmental island with a diverse flora and high productivity. Although this forest provides essential services to the local population, it has suffered 90% anthropogenic reduction in area. Restoration efforts are now getting under way, including discussion as to the most appropriate reference ecosystem to use.

**Methodology/Principal Findings:**

Genetic diversity of tara was studied in the Atiquipa population and over a wide geographical and ecological range. Neither exclusive plastid haplotypes to loma formations nor clear geographical structuring of the genetic diversity was found. Photosynthetic performance and growth of seedlings naturally recruited in remnant patches of loma forest were compared with those of seedlings recruited or planted in the adjacent deforested area. Despite the greater water and nitrogen availability under tree canopy, growth of forest seedlings did not differ from that of those recruited into the deforested area, and was lower than that of planted seedlings. Tara seedlings exhibited tight stomatal control of photosynthesis, and a structural photoprotection by leaflet closure. These drought-avoiding mechanisms did not optimize seedling performance under the conditions produced by forest interception of fog moisture.

**Conclusions/Significance:**

Both weak geographic partitioning of genetic variation and lack of physiological specialization of seedlings to the forest water regime strongly suggest that tara was introduced to lomas by humans. Therefore, the most diverse fragment of lomas is the result of landscape management and resource use by pre-Columbian cultures. We argue that an appropriate reference ecosystem for ecological restoration of lomas should include sustainable agroforestry practices that emulate the outcomes of ancient uses.

## Introduction

The Guidelines and Primer of the Society for Ecological Restoration call upon practitioners of ecological restoration to rediscover the past to determine what needs to be restored at a given site [1]. Frequently, historical studies reveal that ecosystems targeted for ecological restoration have been shaped in part by human drivers [2], [3]. In these ‘socio-ecological’ ecosystems, the human, cultural and historic dimensions should inform both the construction of a reference ecosystem, and the overall process of goal setting [4].

The restoration of desert oases should address the dual nature of these ecosystems, i.e., as centers of biological diversity and endemicity, and also as magnets and focal points for human activity in otherwise forbidding environments. The best known oases are those created by and dependent on local availability of ground or runoff water. Much rarer are those where the main source of water is the deposition of fog droplets. These fog oases include the monsoonal mountains of the southern Arabian Peninsula [5] and the winter-spring fog oases, locally known as “*lomas*”, found along the coasts of Peru and northern Chile, where the hyperaridity of the Atacama and Peruvian Coastal deserts is punctuated by the interception of thick stratocumulus cloud banks on the sea-facing steep slopes of the coastal ranges [6], [7]. The resulting fogs give rise to an archipelago of nearly 70 discrete lomas inhabited by ca. 1400 plant species with diverse biogeographical affinities. There are many endemic taxa, often exceeding 40% of the local flora, many cases of montane Andean disjunctions, Northern Hemisphere desert disjunctions, and pantropical species as well [8], [9]. The origin of the lomas dates back no more than 4 My, coinciding with a sharp increase in aridity [10]. It has been suggested that forest persistence was due to the efficacy with which the largest trees intercept fog [11].

As foci for human activity, historical, palaeoecological, and archaeological evidence suggest that Incas practiced agroforestry with different arboreal taxa and with water-harvesting techniques, from ca. AD 1100 onwards [12]. Indeed it appears that the Incas, and other native Amerindian peoples in dry parts of South America, planted trees as a land-management practice. These afforestation measures were probably intended to optimize water use and improve crop, animal, and forest production under a climatic regime characterized by scant and seasonal rainfall. Various multipurpose native legume trees were apparently used in this way, including *Acacia*, *Geoffroea*, *Prosopis*, among others [13], [14].

Among the surviving lomas, only Atiquipa (Arequipa province, southern Peru) supports a substantial stand of forest (1260 ha), which has long been recognized as the largest, most diverse and productive of all loma formations [15], [16]. Extensive archaeological remains attest to a major Inca settlement [17], although remains of the earliest human activity in the territory date back to 12500 BP [18]. Today, only ca. 450 people live in the Atiquipa lomas. By the end of the 20^th^ century, this community experienced critical levels of poverty when severe deforestation resulted in water shortages that threatened subsistence agriculture [19], [20].

**Figure 1:**
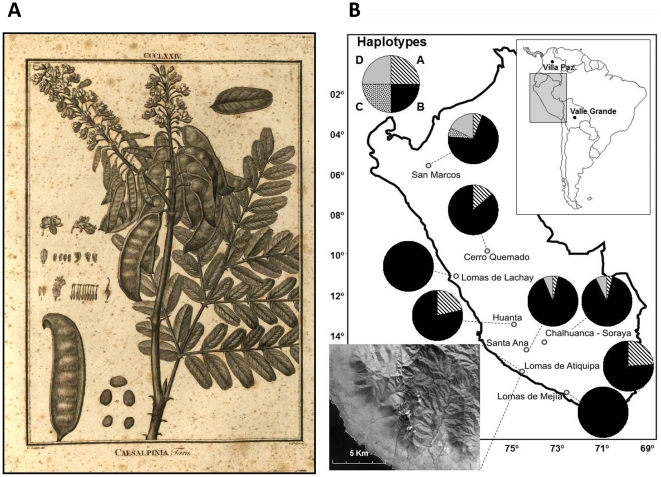
Study species and sample populations. (**A**) Depiction of leaves and reproductive organs of *Caesalpinia spinosa* (taken from the report of Ruíz and Pavón's expedition, 1807–1808). (**B**) Map of Peru, displaying distribution and frequency of cpDNA haplotypes in *Caesalpinia spinosa* populations. Upper Inset: Location of the main map area, and of the Colombian and Bolivian populations. Lower Inset: Lomas de Atiquipa surrounded by the coastal Pacific desert; image from Google Earth™.

Recently, growing awareness of the link between forest conservation and human subsistence in the lomas has promoted forest restoration initiatives [21]. As for many other attempts to restore tropical and subtropical forests elsewhere [22], these efforts consisted primarily in the establishment of tree plantations. In this case, the dominant tree species, tara (*Caesalpinia spinosa* (Mol.) Kuntze; Fabaceae; Fig. 1), was planted to provide both ecological services (mainly watershed protection and direct water supply) and commercial products with an increasing international demand (i.e. tannins and gums) [23], [24].

The aim of the present study was to provide knowledge and insight to help construct appropriate and meaningful ecological reference systems for guiding restoration of the forest ecosystem of the Atiquipa lomas. In this scenario, identification of references is hampered by the lack of written records left by pre-Columbian cultures, and by the inadequacy of palynological analysis of sediment cores to ascertain past occurrence in insect-pollinated species, such as tara. This leaves a critical question unanswered: Is the tara-dominated forest in Atiquipa the result of past human activity? In the present study, we hypothesize that, if anthropogenic influence were negligible, the Atiquipa population of tara would have undergone genetic divergence from other Peruvian populations. We expect this, first, because isolation in environmental islands creates opportunities for adaptive evolution [25]; second, because the large population size of this dominant species can be expected to have favored local adaptation [26]; and third, because significant genetic differentiation has been found in populations of other woody species from Arabian fog oases [5], which suggests that the same could have happened in the South American lomas. We also hypothesize that the Atiquipa population of tara would show functional specialization to the periodic water pulses of the fog oases. Firstly, because, as a drought-deciduous perennial, tara is expected to use specific water sources at certain times of the year, rather than expressing a generalist strategy, absorbing water whenever and wherever it is available [27]. Secondly, because fog in arid environments often promotes morphological and functional specialization [28], [29]. This is highly relevant for restoration purposes, as specialization to fog water pulses may affect plant response to artificial watering during plantation establishment in deforested areas. Testing the proposed hypotheses will help improve the restoration procedures, from seed provenance selection to the appraisal of favorable recruitment conditions. The ultimate goal, however, is to aid construction of a reference ecosystem and, more specifically, to determine whether remnant patches of pristine forest should be preserved in sanctuaries or, on the contrary, certain human activity is required to maintain this forest.

## Results

### Genetic diversity estimated with cpDNA microsatellites

All tested plastid primer pairs yielded amplified products for every sample. All PCRs produced a single major, robust band per primer pair and therefore there was no evidence for heteroplasmy. Sequences of the pilot study of cpDNA regions were deposited in GenBank (HQ011825–HQ011843). No sequence variation was found in the extended sample using the 21 primer pairs (Supporting Information S1), except for size variation at four loci. Accordingly, size variation for the amplified products was only observed for two *ccSSR* and two *cpSSR* loci. The combination of the two alleles found at each of these polymorphic plastid microsatellite loci produced a total of five haplotypes, designated A-E (Tables 1 and 2). Overall, there was no noticeable pattern that related haplotype distribution to geographical or ecological areas (Fig. 1). None of the scored haplotypes was exclusive to any of the study fog oases (loma formations), nor even to this type of habitat. Private haplotypes were only found in the northernmost population, San Marcos (Cajamarca). Strikingly, all the Peruvian populations shared the most frequent haplotype (B) but none of the Peruvian haplotypes was found in Colombia or Bolivia. An analysis of molecular variance (AMOVA) for the Peruvian populations showed that 93% of total genetic diversity occurred within populations (Φ = 0.232, *df* = 170, *P*<0·001), while only 7% was attributable to variation among populations (Φ = 0.019, *df* = 8, *P*<0.001). DNA sequencing primers did not show any further polymorphism.

**Table 1:** Sample localities and haplotype frequencies of the study populations of *Caesalpinia spinosa* (see Table 2 for haplotype characteristics).

*Sample Localities*	*Geographic coordinates*	*Altitude* (m asl)	*Environment* 1	*Haplotypes (N)*
Cerro Quemado, Huanuco, Peru	09°49′36″S, 75°47′55″W	1600	Premontane tropical thorn woodland	A (3) B (19)
Chalhuanca-Soraya, Apurímac, Peru	14°12′19″S, 73°19′26″W	2700	Lower montane subtropical dry forest	A (1) B (25) D (2)
Huanta, Ayacucho, Peru	12°53′35″S, 74°19′08″W	2200	Lower montane subtropical thorn steppe	A (3) B (11)
Lomas de Atiquipa, Arequipa, Peru	15°45′40″S, 74°22′14″W	800	Lomas^2^	A (6) B (19)
Lomas de Lachay, Lima, Peru	11°21′25″S, 77°22′02″W	500	Lomas	B (13)
Lomas de Mejía, Arequipa, Peru	16°46′25″S, 72°14′49″W	700	Lomas	B (29)
San Marcos, Cajamarca, Peru	05°51′36″S, 78°33′42″W	1800	Lower montane tropical dry forest	A (1) B (12) C (1) D (3)
Santa Ana, Ayacucho, Peru	14°43′37″S, 74°07′34″W	2400	Lower montane subtropical thorn steppe	A (1) B (28) D (2)
Villa Paz, Boyacá, Colombia	05°37′41″N, 73°33′46″W	2084	Lower montane dry forest	E (38)
Valle Grande, Santa Cruz, Bolivia	18°20′10″S, 64°08′34″W	1658	Subtropical dry forest	E (34)

1 According to Holdridge life zones system [76].

2 Study lomas are considered as lower-montane warm-temperate/subtropical desert scrubs in the Holdridge's classification.

**Table 2:** Lengths (in bp) of the polymorphic fragments and haplotype composition in *Caesalpinia spinosa*.

*Haplotypes*	*ccSSR-5*	*ccSSR-9*	*cpSSR3*	*cpSSR6*
A	278	173	91	99
B	278	173	92	100
C	277	174	91	99
D	277	174	92	100
E	278	174	92	100

### Environment and seedling performance

In the fog oasis, PAR was reduced by ca. 60% during the fog season of 2007 (Supporting Information S1). This reduction in light intensity was accompanied by a rise in air relative air humidity, with similar values both in the forest and in the adjacent reforested area. Maximum soil water contents were reached, 3 months after the fog had settled. As expected, the highest soil water contents were recorded under the canopy of adult tara trees within the forest. The lowest values of water availability were recorded within the forest gaps, likely due to the combined effect of dense herbaceous vegetation and the lack of fog interception by the tree canopy cover. The soil within the forest exhibited differentially high contents of organic mater, nitrogen, carbon, and iron (Table 3).

**Table 3:** Mean (±1 SD, *n* = 8) and permutation-test *P* values for differences in soil characteristics between the forest and the adjacent reforested area.

	*Remnant Forest*	*Reforested Area*	*P Value*
pH	4.88±0.18^a^	5.09±0.34^a^	0.1728
Conductivity (µs cm^−1^)	303.73±240.89^a^	156.58±156.37^a^	0.1920
Organic matter (%)	3.18±0.81^a^	1.68±0.67^b^	0.0023
N (%)	0.31±0.13^a^	0.11±0.05^b^	0.0007
C (%)	1.85±0.47^a^	0.97±0.39^b^	0.0023
Phosphorus (µg g^−1^)	47.14±31.47 ^a^	58.13±53.25 ^a^	0.6629
Calcium (mg g^−1^)	2.01±0.48^a^	1.76±0.70^a^	0.4694
Iron (µg g^−1^)	31.93 (6.90^a^	24.44±4.64^b^	0.0197
Manganese (µg g^−1^)	75.86±23.92^a^	67.38±19.81^a^	0.4592
Magnesium (µg g^−1^)	304.86±85.86^a^	434.25±193.15^a^	0.1346
Potassium (µg g^−1^)	280.0±192.16^a^	270.25±187.92^a^	0.9210
Sodium (µg g^−1^)	67.43±16.41^a^	80.25±34.24^a^	0.4403

Values across rows with different superscript letters indicate that means were significantly different (Tukey's HSD test, *P*<0.05).

Distance between recruited seedlings and adult trees within the forest was 3-fold shorter than in the reforested area [5.13±2.25 (SD) vs. 17.34±10.07 m, *P*<0.02], reflecting the overall higher tree density in the forest. In consonance, percentage of canopy openness above seedlings was 30% lower in the forest (47.92±11.72 (SD) vs. 68.66±5.45, *P*<0.0001). However, differences in seedling growth (Table 4) were not accounted for by these variables or by vegetation height around seedlings, when considered as covariates. Thus, we found no evidence of enhanced seedling performance under tree canopy. Indeed, seedlings naturally recruited in the forest did not differ in any of the growth parameters measured from those recruited in the adjacent reforested area. Only planted seedlings were significantly taller, leafier and exhibited thicker stems and larger crowns (Table 4). Planted seedlings outperformed naturally recruited seedlings even one year after irrigation was halted, as shown by the longer and thicker internodes produced in that season (Table 4).

**Table 4:** Mean (±1 SD, *n* = 5) and permutation-test *P* values for differences in traits of seedlings either (i) recruited in the forest, (ii) recruited in the adjacent reforested area, or (iii) planted in the reforested area.

	*Remnant Forest*	*Reforested Area*		
*Seedling Trait*	*Recruited*	*Recruited*	*Planted*	*p Value*
Height (cm)	43.80±10.18^a^	49.20±18.40^a^	111.60±38.10^b^	0.0021
Number of tillers	1.60±1.34^a^	1.00±0.00^a^	2.25±0.96^a^	0.4119
Basal stem diameter (mm)	9.43±1.91^ab^	8.86±2.96^a^	15.32±4.48^b^	0.0151
Crown width (cm)	32.60±12.80^a^	26.18±9.25^a^	71.40±27.41^b^	0.0022
Total number of leaves	13.80±7.66^a^	17.40±2.97^a^	51.40±18.72^b^	0.0019
Number of C+1 leaves	4.00±3.54^a^	4.20±2.05^a^	7.20±4.60^a^	0.3079
Number of C+1 internodes	4.20±0.45^a^	4.20±0.45^a^	4.00±0.00^a^	0.9999
C+1 Internode length (mm)	24.51±7.14^a^	33.18±11.95^a^	68.32±21.00^b^	0.0029
C+1 Internode diameter (mm)	4.48±0.63^a^	5.15±0.80^a^	6.70±0.83^b^	0.0031

Leaves and internodes of the previous growth season (C+1) were formed during the year after irrigation of the planted seedlings had ceased.

Values across rows with different superscript letters indicate that means were significantly different (Tukey's HSD test, *P*<0.05).

At the transition from the wet to dry season, the lowest water potentials were recorded at midday in the seedlings recruited within the forest (*P*<0.001, Table 5), in consonance with the significantly lower soil moisture levels (*P*<0.021), and the low water availability at the forest gaps (Supporting Information S1). Percentage of canopy openness above the study seedlings, when included as a covariate, did not account for the variation in soil moisture or leaf water potential. Consistent with this water deficit, SLA was significantly lower in seedlings naturally recruited in the forest than in planted ones (*P*<0.005, Table 5). Variation in SLA was negatively correlated with chlorophyll content on a leaf area basis (*r* = −0.42, *P*<0.001), and, in turn, chlorophyll content was negatively correlated with slight but significant changes in VAZ on a chlorophyll content basis (*r* = −0.78, *P*<0.001, Table 5). This variation in SLA, chlorophylls, and VAZ were not correlated with the large variation observed in canopy openness, nor did they produce differences in photosynthetic performance. Average (±1SE) Amax was 1.79±0.55 µmol m^−2^ s^−1^, ϕ 0.03±0.005 mol mol^−1^, and LCP 31.23±4.02 µmol m^−2^ s^−1^. This low photosynthetic capacity probably reflects a tight control by stomatal conductance, as suggested by the high correlation between Amax and light-saturated stomatal conductance (*r* = 0.96, *P*<0.0001). Structural photoprotection was achieved by leaflet closure, which reduced the leaf area exposed to solar radiation. Leaflet movements took place in concert with environmental variation. Multiple forward stepwise regressions revealed that air relative humidity was the only variable that predicted a significant proportion of the variance in leaflet angle (*r* ^2^ = 0.56, *P*<0.0001). This effect of air relative humidity was consistent across experimental seedling groups, as revealed by the test for homogeneity of slopes (*P*>0.34). The contributions of soil moisture, air temperature, and PAR light intensity were redundant or non-significant.

**Table 5:** Means (±1 SD) and ANOVA *P* values for differences in environmental and physiological features of seedlings either (i) recruited in the forest, (ii) recruited in the adjacent reforested area, or (iii) planted in the reforested area.

	*Remnant Forest*	*Reforested Area*			
*Feature*	*Recruited*	*Recruited*	*Planted*	*p Value*	*n*
Canopy openness (%)	47.92±11.72^a^	66.27±6.46^b^	71.06±3.14^b^	0.0004	6
Soil moisture (%)	14.31±4.13^a^	20.50±3.74^a^	17.64±7.24^a^	0.0207	8
Ψ (MPa)	−2.12±0.43^a^	−1.34±0.45^b^	−1.50±0.48^b^	0.0001	8
SLA (cm^2^ g^−1^)	73.39±14.56^a^	76.31±13.76^ab^	82.94±12.22^b^	0.0160	7
Chl a+b (µmol m^−2^)	211.46±77.41^a^	171.69±62.15^b^	197.21±59.78^ab^	0.0282	7
β-carotene (mmol mol^−1^ Chl)	134.89±28.45^a^	136.77±34.40^a^	129.20±21.48^a^	0.2159	7
VAZ (mmol mol^−1^ Chl)	104.50±38.15^a^	113.89±25.01^a^	107.39±18.04^a^	0.0001	7

*P*-values for midday soil moisture (TDR volumetric percentage) and midday leaf water potential (Ψ) were calculated using Friedman tests with “*Sampling day*” as a within-block factor. *P*-values for specific leaf area (SLA), contents of total chlorophyll (Chla+b), β-carotene, and xanthophyll cycle pool (VAZ) were calculated using a nested ANOVA approach based on pairwise permutations, with plant nested within seedling experimental group.

Values across rows with different superscript letters indicate that means were significantly different (Tukey's HSD test, *P*<0.05).

## Discussion

Our results strongly suggest that the presence and dominance of tara (*Caesalpinia spinosa*) in the Atiquipa fog oasis is attributable to past human activity. This implies that the current configuration of the most diverse and heavily forested fragment of loma forest in the archipelago of Peruvian fog oases is in part the result of pre-Columbian human activities, including the introduction, selection and planting of the multi-purpose tara tree. This conclusion is supported by the weak geographical partitioning of genetic variation among tara populations, and by the poor tara seedling recruitment, performance and growth within the loma forest of Atiquipa. Furthermore, there is clear evidence for this kind of ecosystem management practice elsewhere in the region during the Inca Empire [12].

The low level of genetic differentiation among the Peruvian populations shown by AMOVA may be a reflection of interpopulation gene flow. Historical accounts describe more extensive forests [30] the connectivity of which may have been enhanced by wet episodes associated with El Niño Southern Oscillation (ENSO) events [9]. However, even on the assumption of historical habitat integrity, gene flow would have been limited by phenological barriers. Optimal blooming season, when most species were in flower, occurs up to 4 months earlier in the northern than in the southern lomas [31]. The lack of divergence across the wide geographical and environmental range spanned by the study populations contrasts with the high level of genetic differentiation found in another neotropical *Caesalpinia* species along a similar latitudinal gradient [32], and in woody species from other fog oases in the Arabian peninsula [5]. Finally, if tara were a native species of the Atiquipa loma forest, it is likely that its breeding system and dispersal mechanisms would have promoted population divergence [33]. Firstly, this species is most likely pollinated by bees - the most widespread pollination mode among Caesalpinioideae [34] - a syndrome which promotes selfing by geitonogamy [35]. Secondly, seed dispersal of tara would have required ingestion by wild Andean ungulates or camelids, as suggested by its germination requirements [36], [37] and historical records [30], followed by efficient dissemination across the complex loma archipelago [8]. Finally, earthquakes and volcanism in Atiquipa can, arguably, trigger pulses of recruitment [38], particularly considering the ability of this species to resprout from root suckers or from damaged trees. Most wild tree species that share this resprouting capacity also exhibit a highly significant genetic differentiation among populations [39]. In sharp contrast, our results revealed weak geographical partitioning of allelic variation, resulting in a high within-population component of genetic diversity. All of the populations sampled within the limits of the Inca empire shared one haplotype, which was absent from the non-Inca populations of Bolivia and Colombia. This haplotype was dominant in the Atiquipa lomas and the only one detected in the other two loma populations sampled. This pattern, similar to those found in other economically-useful neotropical trees [40], [41] and Andean-cultivated species, has been attributed to the effect of trade between pre-Columbian farmers [42], [43]. The higher genetic diversity found in San Marcos (northern Peru) compared to that beyond the borders of the Inca Empire, may be indicative of proximity to the cultivation centre [42]. Trees vegetatively propagated by man often exhibit a low reduction in genetic diversity relative to wild ancestors [44]. Alternatively, cultivation practices may have locally increased genetic diversity, as farmers enable hybridization between sympatric plants, or introduce new stock from wild populations by trading, or by including landraces for different uses, or simply to hedge their bets [45], [46].

Lack of divergence in populations of tara was consistent with the lack of morphofunctional evidence for ecotypic differentiation in the Atiquipa lomas. Our findings indicate that the seedlings of tara in the Atiquipa loma forest exhibited a conservative drought-avoiding strategy, common in trees from tropical dry forests [47]. Drought avoidance in tara involved a dual regulatory mechanism: tight stomatal control of photosynthesis, and structural photoprotection by leaflet closure. The strong dependence of both responses on relative humidity likely accounted for the lack of differences in growth between seedlings recruited in the forest and in the deforested area, despite the differences found in soil fertility, as fog forms equally in both sites. A high sensitivity to leaf-air vapor pressure deficit is often associated with drought resistance in woody perennials from tropical dry forests [48]. Strikingly, seasonally dry Mesoamerican forests harbor two closely related species of tara, *C. cacalaco* and *C. vesicaria* [49], which suggests a common primary habitat. Besides, these species are pioneers in open communities, which coincides with the early successional behavior of tara, as shown by the high-light requirements for successful establishment of their seedlings [cf. 50]. Forest dominance by early successional tree species has been frequently attributed to human activity [51].

Local acclimation, suggested by SLA, and leaf contents of chlorophylls and xanthophylls, was not primarily driven by tree canopy cover. This apparent lack of local specialization to periodic water pulses under a fog-trapping overstory might be an artifact owing to the small sample size. However, human introduction is further supported by the differential seedling growth response. Planted taras outperformed naturally recruited seedlings of the same age, likely due to nursery-improved early growth [52]. In these planted seedlings, initial watering apparently did not trigger phenotypic responses, such as shallow root differentiation or vascular adjustments, which may be detrimental after irrigation offset [27]. In contrast, tara exhibits a suite of drought-avoiding mechanisms that appears to facilitate plantation in other multi-purpose, dry-tropical-forest tree legumes (e.g. *Acacia* spp.) [53], and has been considered a generalist strategy favored by human activities in arid ecosystems [54]. Tara may well have been deliberately spread by the Inca as a source of dye and tannins [55], and/or unintentionally, when the pods were grazed by domestic camelids [30]. Tara could have been introduced before - but not after - Inca times, as documented by the Jesuit scholar Bernabé Cobo in 1620 [15], and by archaelogical findings, such as calabashes or gourd-shells containing tara leaves at the Incan site of Chuquitanta (Lima) [14].

This is the first time, to our knowledge, that elaboration of an ecosystem of reference to guide restoration programmes has been addressed experimentally, in the context of the eco-cultural restoration of oases of any kind. Considering its putative anthropogenic introduction in the lomas, tara might be excluded from the loma reference ecosystem in an attempt to recover “wilderness”. Several lines of evidence, however, suggest that this would be an oversimplified view of the human interactions with this landscape and an inappropriate way to orient a restoration program. Tree species of the loma forest (*C. spinosa*, *Myrcianthes ferreyrae*, *Acacia macracantha*, *Carica candicans*, *Hesperomeles lanuginosa*) are all useful for people as sources of non-timber forest products. Local columnar cacti (*Echinopsis* spp.) were also likely used and managed by the Inca, in this case because of the psychotropic properties of their sap [56].

We agree with Chepstow-Lusty & Winfield [12], who argued that ecological restoration strategies in the Andean region should be formed in the light of emerging evidence of sophisticated Inca land management practices, sometimes called the Inca model. Also known as the “vertical archipelago”, the Inca model was based on ecological complementarity, that is, on the simultaneous control or manipulation of multiple ecological tiers along altitudinal gradients [57]. In Atiquipa, this segregation in resource use was enabled by the outstanding Inca achievements in hydraulic engineering which diverted fog water collected by the loma forest to irrigate areas (“*andenerías*”) at lower altitudes [58]. Loma forest was considered a water source and storage area, and, thus, was mainly devoted to mixed forestry and camelid rearing [58], and only to a lesser extent to smallholding agriculture [30]. In this historical context, we surmise that our findings do not merely suggest an anthropogenic cause for the presence and dominance of tara in the loma forest, but also provide evidence of this fog-oasis ecosystem persisting as part of a socio-ecological system. In conclusion, we suggest that restoration models of the unique and highly threatened loma ecosystem should incorporate the implementation of sustainable practices that emulate the outcomes of ancient uses. In this way, the presumed socio-ecological character of these formations would be perpetuated – or reconstituted where it has been lost – and the resulting model would also be far more attractive to local people that should be integrated into the ecological restoration program. As noted, direct economic justification for the project can be found not only in the ecosystem services provided by the lomas, and their cultural significance, but also by the fact that landowners and municipalities in Peru, and Ecuador, are increasingly working to develop and exploit a growing international market for tara pods in the agroalimentary industry [23], [24].

The importance of the present study lies in the demonstration, from a biological perspective, of the relevance of a holistic approach to ecological restoration in an oasis setting, but, obviously, deeper genetic and ecophysiological studies are needed for a further understanding of tara behavior in the Peruvian loma fragments and in lomas undergoing experimental restoration.

## Materials and Methods

### Population genetic analyses

Eight tara populations were intentionally chosen to encompass the environmental range of this Andean tree in Peru, across a latitudinal gradient approximately 1500 km long (Fig. 1; Table 1). We also sampled one population in Colombia, about 500 km north of the northernmost border of the Inca Empire, and one in Bolivia, about 200 km south of the southernmost Peruvian study population, and 100 km east of the easternmost border of the Inca Empire (according to [59]). We analyzed 13–38 individuals per population based on availability of trees at least 10 m apart, with at least 5 m in height and with dbh (diameter at breast height) greater than 10 cm. Fresh leaves were collected from each individual and dried in silica gel *in situ*. Isolation of DNA followed the protocol accompanying the DNeasy Plant Mini Kit (Qiagen Inc, Hilden, Germany). We tested 21 primer pairs to identify polymorphic plastid sequence variation (Supporting Information S1). Eight of these were universal angiosperm primers developed for tobacco [60], [61]. Six plastid primers were already found to be hypervariable within species of land plants [62]. Three were primer pairs designed for *Caesalpinia echinata* Lam. [32]. The last four were species-specific primer pairs designed from the nucleotide sequence of the *trn*H(GUG)-*trn*K(UUU), the *trn*Q(UUG)-*rps*16, the *trn*S(GCU)-*trn*G(UCC), and the *trn*S(UGA)-*trn*fM(CAU) regions of the *C. spinosa* plastid genome. PCR conditions are described in Supporting Information S1.

### Study site and sampling locations

Seedling performance was assessed at the Atiquipa lomas in the District of Atiquipa, Department of Arequipa, Peru, located on a steep altitudinal gradient that ranges from the sea level to an altitude of 1297 m.a.s.l. in less than 20 km (Fig. 1). Within the coastal desert, this site constitutes a biogeographical and environmental island separated by more than 100 km from any other forested area. In this oasis, fog trapped by vegetation is the main water influx. In the Peruvian coastal lomas, annual throughfall under tree canopy has been reported to exceed 500 mm [6], whereas annual precipitation in Atiquipa is an order of magnitude lower (59 mm, 1966–80) [63]. Historical deforestation on the study site resulted in landscape transformation and fragmentation with extensive areas of seasonal grasslands surrounding the last remnants of the loma forest. Nowadays, the loma forest at Atiquipa occupies only 1260 ha, approximately one-tenth of the original area, which roughly coincides with the reduction undergone by the Peruvian lomas as a whole (from 15000 to 2000 km^2^) [64].

Density of adult tara trees, averaged across three 50×50 m plots per area, was five times greater in the loma forest than in the adjacent deforested area [117±67 (SD) vs. 24±18 trees/ha]. In July–August 2003, tara plantations were established on the deforested area. Five-month-old seedlings, nursery-raised from local seeds, were hand-planted on a 9×9 m grid. Goat manure (500 mg) was added as an organic fertilizer to each planting hole (30×30×40 cm). After planting, seedlings were hand-watered weekly with 7 l of water, for two years. Irrigation water was previously collected by fog catchers during fog events, immediately conveyed via underground lines to covered reservoirs, and then distributed to storage tanks in the plantation plots [21]. Stand density was around 150 trees/ha at the time growth and ecophysiological measurements were made.

Air temperature and relative humidity 1 m above the soil surface, as well as photosynthetically active radiation (PAR, 400–700 nm) levels were recorded every 30 min for 1 year, from November 2007 to November 2008, with microclimatic sensors (HOBO, Onset Computers, Pocasset, MA, USA) in a gap within the forest and at the adjacent reforested area. In these two sites, we simultaneously monitored soil water content variation at a depth of 10 cm by microclimatic sensors (HOBO Soil Moisture smart sensor, Pocasset, MA, USA) placed beneath and outside the canopy of an adult tara tree.

### Morphological and ecophysiological measurements

Natural recruitment of tara was found to be extremely low both within the forest and the adjacent reforested area (Supporting Information S1). Biological and logistic constraints limited the number of available replicates. In each of these two habitats, we selected 8 recruited seedlings of the same age as those planted in 2003. As tara is able to resprout from root suckers, soil was carefully explored around every plantlet to confirm its origin from seed. We then selected the nearest planted seedling to each of the 8 seedlings chosen among those naturally recruited in the reforested area. Seedlings with signs of past breakage or herbivory damage were discarded. Soil samples to a depth of 10 cm were collected in the forest, in the immediate vicinity of the selected seedlings, and in the reforested area, from locations halfway between the selected recruited and planted seedlings (8 samples×2 habitats). Each soil sample was analyzed separately by standard procedures (Supporting Information S1). In November 2007, at the transition from the wet to dry season, we analyzed the growth of 5 seedlings from each experimental group (i.e. recruited within the forest, recruited in the reforested area, or planted). We specifically measured seedling height, number of tillers, basal stem diameter, crown width, total number of leaves, number of leaves from previous season's cohorts, surrounding vegetation height and distance to the closest adult tree. Lengths and diameters of the internodes formed during the previous season were measured to assess the effect of irrigation cessation (July 2005) on planted seedlings by comparison with the growth of those recruited naturally.

A field ecophysiological survey was carried out in November 2007. Soil moisture and leaf water potential were measured at midday (1300–1600 h local time) using, respectively, a portable TDR (HH2, Delta-T, Burwell, Cambridge, U.K.) and a Scholander-type pressure chamber (SKPM 1400, Skye Instruments Ltd., Llandrindod Wells, U.K.). These measurements were taken in 8 seedlings per experimental group and were repeated in two consecutive clear days. Specific leaf area (SLA) and contents of photoprotective pigments [i.e. xanthophyll cycle pigments (VAZ) and β-carotene on a chlorophyll content basis] were determined in three fully-expanded leaflets from 7 seedlings for each experimental group. Leaflet discs were collected and stored in paper envelopes filled with silica gel until extraction and transported to the analytical laboratory [65]. Photoprotective pigment pools were separated by HPLC (Waters Corp., Milford, MA, USA), following pigment extraction in cool acetone. In order to assess the shelter provided by the canopy of adjacent trees in each individual seedling, hemispheric photographs were taken immediately above the seedling crowns with a digital camera Nikon Coolpix 4500 (Nikon Canada Inc., CA) coupled with a Nikon FC-E8 Fisheye Adapter (Nikon, Japan). All the photographs were taken before sunset or under uniformly cloudy conditions and analyzed with Gap Light Analyzer software v2.0 to estimate the percentage of canopy openness. Carbon assimilation response to light was characterized in 4 plants randomly selected among those sampled for growth and ecophysiological analyses within each experimental group. Then, light curve parameters were averaged across groups to obtain a more representative estimation of photosynthetic performance (*n* = 12). Net carbon assimilation was recorded with a Li-Cor 6400 infrared gas analyzer (LiCor Inc., Lincoln, NE, USA) in the field (Supporting Information S1). Finally, structural photoprotection was assessed by measuring leaflet angle. As leaflets of the study species can move in response to environmental stimuli, we measured the angle to the horizontal of a single leaflet per leaf, in 10 different leaves per plant, in 8–10 plants per group, from 0830 to 1400 h local time. At each plant, we simultaneously recorded soil moisture, and air temperature, relative humidity, and solar (PAR) light intensity at zenith angle above canopy level.

### Data analysis

Differentiation among Peruvian populations at microsatellite loci was assessed by an analysis of molecular variance (AMOVA) with ARLEQUIN, v 2.00 [66]. Differences in environmental, morphological and ecophysiological variables were tested by permutation methods, as recommended for small sample sizes [67], [68]. Permutation tests have equal or higher power than those based on normal theory [69]. Differences in soil characteristics and plant growth between experimental groups were compared using one-way sample permutation tests. Effect of canopy cover was controlled for by incorporating surrounding vegetation height, distance to the closest adult tree, or percentage of canopy openness as individual covariates in maximally selected statistics tests [70]. Differences between experimental groups in soil moisture and leaf water potential were evaluated using Friedman tests incorporating sampling day as a within-block factor. All tests were computed using the package “coin” in R [71]–[73] approximating the null distribution of the test statistic by Monte-Carlo resampling with 100000 replications. Differences between plants and experimental groups in SLA and pigment contents were determined using a nested ANOVA approach (plant nested within group), using the function “aovp” of the package “lmPer” [74]. This approach is analogous to a conventional ANOVA except that p-values are obtained by pairwise permutation of the data instead of being derived from F-tests [74]. Light curves were fitted by nonlinear regression using the Mitscherlich model equation. The variance explained by this model was very high [mean *r* ^2^ = 0.97±0.02 (SD)]. The light-saturated rate of photosynthesis (Amax) was provided by the asymptote of the function, the apparent quantum yield (φ) by the initial slope of the curve, and the light compensation point (LCP) by the x-intercept [75]. A forward stepwise multiple regression was used to elucidate the contribution to the variation in leaflet angle of soil moisture, PAR light intensity, and air temperature and relative humidity, with *P*<0.05 set as the inclusion criterion. Interactions between these predictors and the categorical factor (seedling experimental group) were tested (test for homogeneity of slopes) to check for confounding effects.

## Supporting Information

Supporting Information S1 **Appendix S1.** Additional details of Material and Methods. **Figure S1.** Annual microclimatic patterns in the forest and at the adjacent reforested area. **Table S1.** Primer sequences used in the analyses of plastid DNA.
(DOC)

## References

[1] SER (2004). The SER *International Primer on Ecological Restoration*.

[2] Aronson J, Dhillion S, Le Floc'h E (1995). On the need to select an ecosystem of reference, however imperfect: a reply to Pickett and Parker.. Restor Ecol.

[3] Young O, Berkhout F, Gallopin GC, Janssen MA, Ostrom E (2006). The globalization of socio-ecological systems: An agenda for scientific research.. Global Environ Chang.

[4] Egan D, Howell EA (2001). The Historical Ecology handbook. A restorationist's guide to reference ecosystems.

[5] Meister J, Hubaishan MA, Kilian N, Oberprieler C (2005). Chloroplast DNA variation in the shrub *Justicia areysiana* (Acanthaceae) endemic to the monsoon affected coastal mountains of the southern Arabian Peninsula.. Bot J Linn Soc.

[6] Péfaur JE (1982). Dynamics of plant communities in the lomas of southern Peru.. Vegetatio.

[7] Pinto R, Barría I, Marquet PA (2006). Geographical distribution of *Tillandsia* lomas in the Atacama Desert, northern Chile.. J Arid Environ.

[8] Dillon MO, Nakazawa M, Leiva S, Haas J, Dillon MO (2003). The lomas formations of coastal Peru: composition and biogeographic history.. El Niño in Peru: biology and culture over 10,000 years.

[9] Dillon MO, Hollowell V, Keating T, Lewis W, Croat T (2005). Solanaceae of the lomas formations of coastal Peru and Chile.. A Festschrift for William G. D'Arcy: The Legacy of a Taxonomist.

[10] Huertas ML, Schneider JV, Zizka G (2007). Phylogenetic analysis of *Palaua* (Malveae, Malvaceae) based on plastid and nuclear sequences.. Syst Bot.

[11] Villagrán C, Armesto JJ, Hinojosa LF, Cuvertino J, Pérez C, Squeo FA, Gutiérrez JR, Hernández IR (2004). Capítulo 1. El enigmático origen del bosque relicto de Fray Jorge.. Historia Natural del Parque Nacional Bosque Fray Jorge.

[12] Chepstow-Lusty A, Winfield M (2000). Inca agroforestry: lessons from the past.. Ambio.

[13] Aronson J (1990). Desert plants of use and charm from northern Chile.. Desert Plants.

[14] Towle M (2007). The Ethnobotany of pre-Columbian Peru.

[15] Cobo B (1956). [1653] Historia del Nuevo Mundo.

[16] Vázquez de Espinosa A (1992). [1629] Compendio y descripción de las Indias Occidentales. Crónicas de América, vol. 68.. Madrid: Historia.

[17] Linares E (1990). Prehistoria de Arequipa. Tomo II.

[18] Keefer DK, deFrance SD, Moseley ME, Richardson JB, Satterlee DR (1998). Early maritime economy and El Niño events at Quebrada Tacahuay, Peru.. Science.

[19] Masuda S, Masuda S, Shimada I, Morris C (1985). Algae collectors and lomas.. Andean ecology and civilization.

[20] Ferreyra R, Mejía Baca J (1986). Flora y vegetación del Perú.. Gran geografía del Perú: naturaleza y hombre, vol. 2.

[21] Torres J, Velásquez D, Sivakumar MVK, Ndiang'ui N (2007). Successful experiences of sustainable land use in hyperarid, arid and semiarid zones from Peru.. Climate and Land Degradation.

[22] Lamb D, Erskine PD, Parrotta JA (2005). Restoration of degraded tropical forest landscapes.. Science.

[23] de la Cruz P (2004). Aprovechamiento integral y racional de la tara *Caesalpinia spinosa* - *Caesalpinia tinctoria*.. Rev Inst Inv FIGMMG.

[24] Villanueva C (2007). La tara. El oro verde de los incas para el mundo.

[25] Ackerly DD (2003). Community assembly, niche conservationism, and adaptive evolution in changing environments.. Int J Plant Sci.

[26] Leimu R, Fischer M (2008). A meta-analysis of local adaptation in plants.. PLoS ONE.

[27] Schwinning S, Ehleringer JR (2001). Water use trade-offs and optimal adaptations to pulse-driven arid ecosystems.. J Ecol.

[28] Martorell C, Ezcurra E (2007). The narrow-leaf syndrome: a functional and evolutionary approach to the form of fog-harvesting rosette plants.. Oecologia.

[29] Simonin KA, Santiago LS, Dawson TE (2009). Fog interception by *Sequoia sempervirens* (D. Don) crowns decouples physiology from soil water deficit.. Plant Cell Environ.

[30] Rostworowski M (1981). Recursos Naturales Renovables y Pesca, Siglos XVI y XVII.

[31] Ferreyra R (1983). Los tipos de vegetación de la costa peruana.. An J Bot Madrid.

[32] Lira CF, Cardoso SRS, Ferreira PCG, Cardoso MA, Provan J (2003). Long-term population isolation in the endangered tropical tree species *Caesalpinia echinata* Lam. revealed by chloroplast microsatellites.. Mol Ecol.

[33] Hamrick JL, Godt JW (1996). Effects of life history traits on genetic diversity in plant species.. Philos T Roy Soc B.

[34] Borges LA, Sobrinho MS, Lopes AV (2009). Phenology, pollination, and breeding system of the threatened tree *Caesalpinia echinata* Lam. (Fabaceae), and a review of studies on the reproductive biology in the genus.. Flora.

[35] Eynard C, Galetto L (2002). Pollination ecology of *Geoffroea decorticans* (Fabaceae) in central Argentine dry forest.. J Arid Environ.

[36] Teketay D (1996). Germination ecology of twelve indigenous and eight exotic multipurpose leguminous species from Ethiopia.. Forest Ecol Manag.

[37] Rossini S, Valdés B, Andrés MC, Márquez F, Bueso M (2006). Germinación de las semillas en algunas especies americanas de Fabaceae y Bignoniaceae cultivadas en Sevilla (SO España).. Lagascalia.

[38] Vittoz P, Stewart GH, Duncan RP (2001). Earthquake impacts in old-growth *Nothofagus* forests in New Zealand.. J Veg Sci.

[39] Smith S, Hughes J, Wardell-Johnson G (2003). High population differentiation and extensive clonality in a rare mallee eucalypt: *Eucalyptus curtisii*.. Conserv Genet.

[40] Loveless MD, Gullison RE, Lugo AE, Figueroa-Colon JC, Alayon M, Big-leaf mahogany: genetics, ecology, and management. Editors (2003). Genetic variation in natural mahogany populations in Bolivia.. Ecological Studies 159.

[41] Shepard GH, Ramirez H (2011). “Made in Brazil”: human dispersal of the Brazil nut (*Bertholletia excelsa*, Lecythidaceae) in ancient Amazonia.. Econ Bot.

[42] Zimmerer KS, Douches DS (1991). Geographical approaches to crop conservation: the partitioning of genetic diversity in Andean potatoes.. Econ Bot.

[43] Papa R, Gepts P (2003). Asymmetry of gene flow and differential geographical structure of molecular diversity in wild and domesticated common bean (*Phaseolus vulgaris* L.) from Mesoamerica.. Theor Appl Genet.

[44] Miller AJ, Schaal BA (2006). Domestication and the distribution of genetic variation in wild and cultivated populations of the Mesoamerican fruit tree *Spondias purpurea* L. (Anacardiaceae).. Mol Ecol.

[45] Romão RL (2000). Northeast Brazil: A secondary center of diversity for watermelon (*Citrullus lanatus*).. Genet Res Crop Evol.

[46] Parker KC, Trapnell DW, Hamrick JL, Hodgson WC, Parker AJ (2010). Inferring ancient *Agave* cultivation practices from contemporary genetic patterns.. Mol Ecol.

[47] Sandquist DR, Cordell S (2007). Functional diversity of carbon-gain, water-use, and leaf-allocation traits in trees of a threatened lowland dry forest in Hawaii.. Am J Bot.

[48] Olivares E, Medina E (1992). Water and nutrient relations of woody perennials from tropical dry forests.. J Veg Sci.

[49] Simpson BB, Larkin LL, Weeks A, Klitgaard BB, Bruneau A (2003). Progress towards resolving the relationships of the *Caesalpinia* group (Caesalpinieae: Caesalpinioideae: Leguminosae).. Advances in legume systematics, part 10.

[50] Poorter L (2007). Are species adapted to their regeneration niche, adult niche, or both?. Am Nat.

[51] D'Orangeville L, Bouchard A, Cogliastro A (2008). Post-agricultural forests: Landscape patterns add to stand-scale factors in causing insufficient hardwood regeneration.. Forest Ecol Manag.

[52] Villar-Salvador P, Planelles R, Enríquez E, Peñuelas Rubira J (2004). Nursery cultivation regimes, plant functional attributes, and field performance relationships in the Mediterranean oak *Quercus ilex* L.. Forest Ecol Manag.

[53] Gebrekirstos A, Teketay D, Fetene M, Mitlöhner R (2006). Adaptation of five co-occurring tree and shrub species to water stress and its implication in restoration of degraded lands.. Forest Ecol Manag.

[54] Haase P, Pugnaire FI, Clark SC, Incoll LD (2000). Photosynthetic rate and canopy development in the drought-deciduous shrub *Anthyllis cytisoides* L.. J Arid Environ.

[55] Barreiro RPAJ (1931). Relación del viaje hecho a los reynos del Perú y Chile por los botánicos y dibuxantes enviados para aquella expedición, extractado de los diarios por el orden que llevó en estos su autor Don Hipólito Ruiz.

[56] Kvist LP, Moraes M (2006). Plantas psicoactivas.. Bot Econ Andes Centr.

[57] Murra JV, Masuda S, Shimada I, Morris C (1985). “El Archipiélago Vertical” revisited.. Andean ecology and civilization.

[58] Canziani J (2007). Paisajes culturales y desarrollo territorial en los Andes.. Cuad Arquitect Ciud.

[59] Conrad GW (1981). Cultural materialism, split inheritance, and the expansion of ancient Peruvian empires.. Am Antiq.

[60] Weising K, Gardner RC (1999). A set of conserved PCR primers for the analysis of simple sequence repeat polymorphisms in chloroplast genomes of dicotyledonous angiosperms.. Genome.

[61] Chung SM, Staub JE (2003). The development and evaluation of consensus chloroplast primer pairs that possess highly variable sequence regions in a diverse array of plant taxa.. Theor Appl Genet.

[62] Shaw J, Lickey EB, Schilling EE, Small RL (2007). Comparison of whole chloroplast genome sequences to choose noncoding regions for phylogenetic studies in angiosperms: the tortoise and the hare III.. Am J Bot.

[63] CIZA, ONERN, SENAMHI (1989). Aprovechamiento de nieblas costeras en las zonas áridas de la costa, lomas de Atiquipa (Prov. Caravelí, Dpto. Arequipa).

[64] Dourojeanni M (1982). Recursos naturales y desarrollo en América Latina y el Caribe.

[65] Esteban R, Balaguer L, Manrique E, Rubio de Casas R, Ochoa R (2009). Alternative methods for sampling and preservation of photosynthetic pigments and tocopherols in leaf samples from remote locations.. Photosynth Res.

[66] Schneider S, Roessli D, Excoffier L (2000). ARLEQUIN ver. 2.00: Software for Population Genetics Data Analysis.

[67] Anderson MJ (2001). Permutation tests for univariate or multivariate analysis of variance and regression.. Can J Fish Aquat Sci.

[68] Kherad-Pajouh S, Renaud O (2010). An exact permutation method for testing any effect in balanced and unbalanced fixed effect ANOVA.. Comput Stat Data An.

[69] Fraker ME, Peacor SD (2008). Statistical tests for biological interactions: A comparison of permutation tests and analysis of variance.. Acta Oecol.

[70] Lausen B, Schumacher M (1992). Maximally selected rank statistics.. Biometrics.

[71] Hothorn T, Hornik K, van de Wiel MA, Zeileis A (2006). A Lego System for Conditional Inference.. Am Stat.

[72] Hothorn T, Hornik K, van de Wiel MA, Zeileis A (2008). Implementing a class of permutation tests: The coin package.. J Stat Softw.

[73] R Development Core Team (2009). R: A language and environment for statistical computing.

[74] Wheeler B (2010). lmPerm: Permutation tests for linear models.. http://CRAN.R-project.org/package=lmPerm.

[75] Potvin C, Lechowicz MJ, Tardiff S (1990). The statistical analysis of ecophysiological response curves obtained from experiments involving repeated measures.. Ecology.

[76] INRENA (1995). *Mapa ecológico del Perú*.

