# Supplementary material for: Forest Restoration in a Fog Oasis: Evidence Indicates Need for Cultural Awareness in Constructing the Reference
Source: PLoS One. 2011 Aug 2;6(8):e23004. doi: 10.1371/journal.pone.0023004 (PMC3149065; doi:10.1371/journal.pone.0023004)
Supplement: Supporting Information S1 — Appendix S1. Additional details of Material and Methods. Figure S1. Annual microclimatic patterns in the forest and at the adjacent reforested area. Table S1. Primer sequences used in the analyses of plastid DNA. (DOC) [file pone.0023004.s001.doc]

**Supporting Information S1**

**Appendix S1.** Additional details of Material and Methods.

STUDY SPECIES

Tara (*Caesalpinia spinosa* (Mol.) Kuntze, Fabaceae) is a drought-deciduous tree species not restricted to lomas (fog oases of the Atacama-Peruvian coastal desert region). It is considered an Andean taxon that thrives in habitats ranging from the desert thorn scrub to the montane dry forest, from nearly sea level to 3150 masl, under arid and semiarid climates of Venezuela, Colombia, Ecuador, Bolivia, Peru and northern Chile and Argentina [1]. Tara is among the few species (less than 6% of loma taxa) known to occur in the northern Peruvian, southern Peruvian, and northern Chilean lomas [2,3].

POPULATION GENETIC ANALYSES

Amplification reactions for microsatellite analyses were performed in a final volumen of 10 μl with 10 ng of template DNA, 0.25 to 0.5 μM of primer labelled either with 6-FAM, HEX or NED fluorophore, 0.5 μM of non-labelled primer, 150 μM of each dNTP (Boehringer Mannheim, Germany), 2.5 mM MgCl_2_, 1x buffer, 0.2 U Ampli*Taq* polymerase (PE Applied Biosystems, Foster City, CA, U.S.A.). The PCR (Polymerase Chain Reaction) was carried out using a GeneAmp PCR System 9700 thermocycler (PE Applied Biosystems, Foster City, CA, U.S.A.). The cycling program consisted of the following steps: 5 min at 94º C, followed by 30 cycles of 30 s at 94 ºC, 30 s at 55 ºC, 45 s at 72 ºC, and a final extension step of 5 min at 72 ºC. Amplification products were resolved onto an automated 310 ABI PRISM DNA sequencer (PE Applied Biosystems, Foster City, CA, U.S.A.), using HD400-ROX as an internal size standard. The PCR fragments were detected with GeneScan analysis software version 3.1 and the alleles were scored using Genotyper DNA fragment analysis software version 2.5.2 (PE Applied Biosystems, Foster City, CA, U.S.A.). Every sample was analyzed at least twice to ensure genotype reproducibility.

Additionally, DNA regions were amplified using the PCR in an Eppendorf Mastercycler Epgradient S (Westbury, NY, U.S.A.). After 1-2 min pretreatment at 94-95ºC, PCR conditions were: 30-40 cycles of 45 s – 1 min at 94-96ºC, 1-2 min at 48-59ºC and 1-2 min at 72ºC. Amplified products were cleaned using spin filter columns (UltraClean PCR Clean-Up Kit, MO BIO Laboratories Inc., CA, U.S.A.). Cleaned products were then directly sequenced using dye terminators (Big Dye Terminator v. 2.0, Applied Biosystems, Little Chalfont, UK) following the manufacturer’s protocols and run into polyacrylamide electrophoresis gels (7%) using an Applied Biosystems Prism model 3700 automated sequencer. Resulting sequence data were assembled and edited using the SeqEd software (PE Applied Biosystems, Foster City, CA, U.S.A.).

SEEDLING RECRUITMENT AND SOIL AND ECOPHYSIOLOGICAL MEASUREMENTS

Seedlings (basal diameter [bd] ≤ 10 mm), saplings (10 mm < bd ≤ 60 mm), and adult trees (bd > 60 mm) were counted along 6 belt-transects (10 x 50 m each), with three of them located in an area more densely forested (194 adult trees ha^-1^) than the other three (47 trees ha^-1^). Seedling densities ranged from 0 to 0.032 m^-2^. The number of seedlings decreased exponentially as the number of adult trees increased (r^2^ = 0.73). Sapling densities followed the same pattern (r^2^ = 0.69) although they were 5 times lower, ranging from 0 to 0,006 m^-2^.

Each soil sample was analyzed separately for pH and soil conductivity and then air-dried and sieved through a 2-mm mesh. Available phosphorus was determined colorimetrically following extraction with calcium and magnesium carbonate, organic carbon and organic matter contents were determined by the Walkley–Black method and nitrogen contents by Kjeldahl analysis. Finally, we assessed calcium, iron, manganese, magnesium, potassium and sodium by Inductively Coupled Plasma Optical Emission Spectroscopy (ICP-OES, Perkin Elmer 4300 DV, Perkin Elmer Corporation, Shelton, CT) after extraction in ammonium acetate at pH 7•0. Net carbon assimilation was recorded at light levels of 2000, 1000, 500, 150, 100, 50, 40, 30, 20, 10 and 0 μmol m^-2^ s^-1^ on one fully-expanded current-year leaf from each plant with a Licor 6400 infrared gas analyzer (LiCor Inc., Lincoln, NE, USA) equipped with a LED-source (LI-6400-02B). Light-saturated stomatal conductance (gs max) was measured at 2000 μmol m^-2^ s^-1^. Cuvette CO_2_ was maintained at 399.2 ± 1.8 μmol mol^-1^ and cuvette air temperature at 20.7 ± 0.5 ºC. Photosynthetic measurements were performed from 0900 to 1300 h under clear to partly cloudy skies on 4 consecutive days. Only three plants (one per experimental group) were measured per sampling morning. The order of experimental groups was alternated each morning.

**Table S1.** Primer sequences used in the analysis of plastid DNA. Some IUPAC symbols are included^1^. Primers generating nucleotide changes in bold.

| ***Forward primer*** | | ***Reverse primer*** | | ***Reference*** |
| --- | --- | --- | --- | --- |
| **ccSSR-5f** | **TCT GAT AAA AAA CGA GCA GTT CT** | **ccSSR-5r** | **GAG AAG GTT CCA TCG GAA CAA** | **4** |
| **ccSSR-9f** | **GAG GAT ACA CGA CAG ARG GAR TTG** | **ccSSR-9r** | **CCT ATT ACA GAG ATG GTG YGA TTT** | **4** |
| ccSSR-14f | GGG TAT AAT GGT AGA TGC CC | ccSSR-14r | GCC GTA GTA AAT AGG AGA GAA A | 4 |
| ccSSR-23f | AYG GRG GTG GTG AAG GGA G | ccSSR-23r | TCA ATT CCC GTC GTT CGC C | 4 |
| CECPSSR1f | TGT TCG TTA TTG TCA AAT CG | CECPSSR1r | TCT TGC TAA TGA TCT AGA TTC A | 5 |
| CECPSSR2f | GCC TAC CCG ATC CAT GTC TA | CECPSSR2r | TTC GGA AAA TTT TTA CCC CC | 5 |
| CECPSSR3f | GAA TCA AAA TTG GAG GAA TTC G | CECPSSR3r | CCA CAA AGG AGA ATT AGG TGA A | 5 |
| **cpSSR3f** | **CAG ACC AAA AGC TGA CAT AG** | **cpSSR3r** | **GTT TCA TTC GGC TCC TTT AT** | **6** |
| **cpSSR6f** | **CGA TGC ATA TGT AGA AAG CC** | **cpSSR6r** | **CAT TAC GTG CGA CTA TCT CC** | **6** |
| cpSSR7f | CAA CAT ATA CCA CTG TCA AG | cpSSR7r | ACA TCA TTA TTG TAT ACT CTT TC | 6 |
| cpSSR10f | TTT TTT TTT AGT GAA CGT GTC A | cpSSR10r | TTC GTC GDC GTA GTA AAT AG | 6 |
| *psb*A | CGA AGC TCC ATC TAC AAA TGG | *trn*H | ACT GCC TTG ATC CAC TTG GC | 7 |
| *trn*E | GGT TCA AGT CCC TCT ATC CC | *trn*F | ATT TGA ACT GGT GAC ACG AG | 8 |
| *trn*H (GUG) | ACG GGA ATT GAA CCC GCG CA | *trn*K (UUU)r | CCG ACT AGT TCC GGG TTC GA | 9 |
| *trn*H(GUG)-polyA | AGT AAA TAA AAA TTG ATG TCTG | *trn*K(UUU) | CCG ACT AGT TCC GGG TTC GA | this study |
| *trn*Lc | CGA AAT CGG TAG ACG CTA CG | *trn*Ld | GGG GAT AGA GGG ACT TGA AC | 8 |
| *trn*Q(UUG) | GCG TGG CCA AGY GGT AAG GC | *rp*S16-polyA | CTA CAT CTT CAA AGT GAC CTC G | this study |
| *trn*S (GCU) | GCC GCT TTA GTC CAC TCA GC | *trn*G (UCC) | GAA CGA ATC ACA CTT TTA CCA C | 7 |
| *trn*S(GCU)-polyA | TTC TAT TAT TAT TTA TTA TTA TTA G | *trn*G(UCC) | GAA CGA ATC ACA CTT TTA CCA C | this study |
| *trn*S (UGA) | GAG AGA GAG GGA TTC GAA CC | *trn*fM (CAU) | CAT AAC CTT GAG GTC ACG GG | 9 |
| *trn*S(UGA)-polyA | ATC CGG TGT GAA TCT ATA GG | *trn*fM(CAU) | CAT AAC CTT GAG GTC ACG GG | this study |

^1^ Y (= C or T), R (= A or G).

**References**

1. de la Cruz P (2004) Aprovechamiento integral y racional de la tara *Caesalpinia spinosa* - *Caesalpinia tinctoria*. Rev Inst Inv FIGMMG: 7: 64–73.
2. Dillon MO, Nakazawa M, Leiva S (2003) The lomas formations of coastal Peru: composition and biogeographic history. In: Haas J, Dillon MO, editors. El Niño in Peru: biology and culture over 10,000 years. Chicago: Field Museum of Natural History. pp. 1—9.
3. Dillon MO (2005) Solanaceae of the lomas formations of coastal Peru and Chile. In: Hollowell V, Keating T, Lewis W, Croat T, editors. A Festschrift for William G. D’Arcy: The Legacy of a Taxonomist. St. Louis: Missouri Botanical Garden Press. pp. 131–155.
4. Chung SM, Staub JE (2003) The development and evaluation of consensus chloroplast primer pairs that possess highly variable sequence regions in a diverse array of plant taxa. Theor Appl Genet 107: 757–767.
5. Lira CF, Cardoso SRS, Ferreira PCG, Cardoso MA, Provan J (2003) Long-term population isolation in the endangered tropical tree species *Caesalpinia echinata* Lam. revealed by chloroplast microsatellites. Mol Ecol 12: 3219–3225.
6. Weising K, Gardner RC (1999) A set of conserved PCR primers for the analysis of simple sequence repeat polymorphisms in chloroplast genomes of dicotyledonous angiosperms. Genome 42: 9–19.
7. Hamilton M (1999) Four primers pairs for the amplification of chloroplast intergenic regions with intraspecific variation. Mol Ecol 8: 521–523.
8. Taberlet P, Gielly L, Pautou G, Bouvet J (1991) Universal primers for amplification of three noncoding regions of chloroplast DNA. Plant Mol Biol 17: 1105–1109.
9. Demesure B, Sodzi N, Petit J (1995) A set of universal primers for amplification of polymorphic non-coding regions of mitochondrial and chloroplast DNA in plants. Mol Ecol 4: 129–134.

**
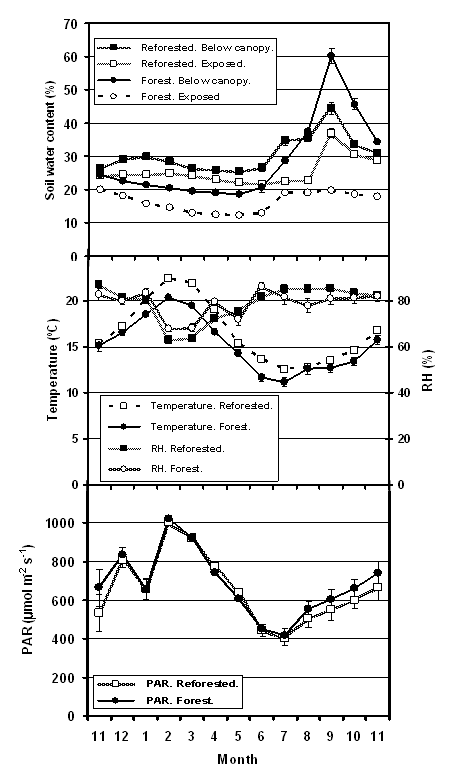
**

**Fig. S1.** Soil water content (%, top), air temperature (ºC, middle), relative humidity (HR, %, middle), and photosynthetic active radiation (PAR, μmol m^-2^ s^-1^, bottom) recorded every 30 minutes and averaged monthly. Microclimate was monitored from November 2007 to November 2008, in a gap within the forest and at the adjacent reforested area. Soil water content at 10-cm depth was measured in both sides beneath and outside the canopy of an adult tara tree. Error bars are standard errors of 28-31 daily means.
